# Identification, Characterization, and Developmental Expression Pattern of Type III Interferon Receptor Gene in the Chinese Goose

**DOI:** 10.1155/2015/186274

**Published:** 2015-05-10

**Authors:** Qin Zhou, Shun Chen, Yulin Qi, Hao Zhou, Mingshu Wang, Renyong Jia, Dekang Zhu, Mafeng Liu, Fei Liu, Xiaoyue Chen, Xue Zhou, Anchun Cheng

**Affiliations:** ^1^Institute of Preventive Veterinary Medicine, Sichuan Agricultural University, Wenjiang, Chengdu, Sichuan 611130, China; ^2^Avian Disease Research Center, College of Veterinary Medicine, Sichuan Agricultural University, Wenjiang, Chengdu, Sichuan 611130, China; ^3^Key Laboratory of Animal Disease and Human Health of Sichuan Province, Sichuan Agricultural University, Wenjiang, Chengdu, Sichuan 611130, China; ^4^Chongqing Academy of Animal Science, Rongchang, Chongqing 402460, China

## Abstract

Interferons, as the first line of defense against the viral infection, play an important role in innate immune responses. Type III interferon (IFN-*λ*) was a newly identified member of IFN family, which plays IFN-like antiviral activity. Towards a better understanding of the type III interferon system in birds, type III interferon lambda receptor (IFNLR1) was first identified in the Chinese goose. In this paper, we had cloned 1952 bp for goose IFNLR1 (goIFNLR1), including an ORF of 1539 bp, encoding a 512-amino acid protein with a 20 aa predict signal peptide at its N terminal and a 23 aa transmembrane region. The predicted amino acid sequence of goIFNLR1 has 90%, 73%, and 34% identity with duck IFNLR1 (predicted sequence), chicken IFNLR1, and human IFNLR1, respectively. And the age-related tissue distribution of goIFNLR1 was identified by Real Time quantitative PCR (RT-qPCR), we found that the goIFNLR1 has a mainly expression in epithelium-rich tissues similar to other species', such as small intestinal, lung, liver, and stomach. Moreover, a relatively high expression of goIFNLR1 was also observed in the secondary immune tissues (harderian gland and cecal tonsil). The identification and tissue distribution of goIFNLR1 will facilitate further study of the role of IFN-*λ* in goose antiviral defense.

## 1. Introduction

Interferons (IFNs), a broad spectrum antiviral agent, act as the first line of defense against viral infection and have an important role in innate immune responses [1]. At present, three distinct types of Interferon are identified which, respectively, are type I, type II, and type III IFNs [2]. Type III interferon (IFN-*λ*) was discovered eleven years ago, in human, which has three subtypes, IFN-*λ*1 (also called IL-29), IFN-*λ*2 (also called IL-28A), and IFN-*λ*3 (also called IL-28B) [3]. IFN-*λ*4 was newly discovered in humans; it is generated by frameshift sub which carried the IFNL4-ΔG allele. And this gene most closely resembles IFN-*λ*3, although their proteins only have 29% amino acid identity, it shares the same signaling pathway with other IFN-*λ* subtypes [4].

The genomic structure of IFN-*λ* is similar to IL-10 family but different from IFNs; however the function of the protein is more closely related to type I interferons than IL-10 [3, 5]. Interestingly, IFN-*λ* was shown to play a nonredundant role in the protection against some virus infection [6]. Virus was recognized by pattern recognition receptors (PRRs) with expression of IFNs which further activated the downstream Jak/STAT signaling pathway via IFN receptors, subsequently inducing the expression of a series of IFN-stimulated genes (ISGs), OAS and Mx1, result in directly hindering the viral replication in host cells [1, 7–9]. Amusingly, type III interferon receptor is a distinct complex, consisting of IFNLR1 (also known as IL-28Ra), which is specific to IFN-*λ*, and IL-10R2 (also known as IL-10Rb) chains, which shared with IL-10-related cytokines [3, 5, 8]. Up till now, type III interferon receptor 1 has been identified in Chicken, Mice, Human, Zebrafish, Cow, Bat, and Toad [3, 10–15]. Different from the immanent receptor of type I IFNs, the tissue distribution of IFNLR1 was restricted and mainly expressed in epithelial cells, which is consistent with the direct immune response in tissues that represent the major sites of virus entry [16–18]. While, IL-10R2 was ubiquitous in body, and has little effect on ligand specificity [18]. The antiviral function of type III IFNs was irreplaceable, probably due to the restricted distribution of IFNLR1 [3, 5, 6, 17].

As known, in mammals, the antiviral activity of IFN-*λ* is mediated through its complexes receptor and primarily acts on epithelia cells [3, 17]. Towards a better understanding of the interaction between goose type III IFN and its receptor, for the first time, the goIFNLR1 cDNA from Sichuan White Goose was identified and the amino acid sequence, structure characterization, and phylogenetic tree of goIFNLR1 were analyzed. Additionally, the tissues expression profiles of goIFNLR1 were investigated in an age-related way. Identification of goIFNLR1 maybe provides the basis for further investigation of the role of goIFN-*λ* in goose antiviral immunity.

## 2. Materials and Methods

### 2.1. Taxonomic Coverage

Thirteen IFNLR1 genes from different bird species, two IFNLR1 genes from reptiles, six IFNLR1 genes from mammals, and one IFNLR1 gene from fish species were chosen as the reference sequences from NCBI GenBank database (http://www.ncbi.nlm.nih.gov/Genbank/) (Table 2).

### 2.2. Animals and Experimental Design

The healthy gooses (the Chinese goose,* Anser cygnoides*) were purchased from the farm of Sichuan Agricultural University (Ya'an city, Sichuan Province). Goslings and adult geese were maintained for 3 days in laboratory animal rooms for acclimatization prior to experiments with water and fodder.

The animal studies were approved by the Institutional Animal Care and Use Committee of Sichuan Agricultural University and followed National Institutes of Health guidelines for the performance of animal experiments.

Eleven kinds of fresh goose tissues, including small intestine (SI), lung (Lu), spleen (SP), liver (Li), kidney (K), cecum (Ce), cecal tonsil (CT), bursa of fabricius (BF), harderian gland (HG), proventriculus (Pr), and thymus (T) from three individual gooses at different age groups, were collected, respectively. The time points for sampling were one-day (1D) group, one-week (1W) group, two-week (2W) group, four-week (4W) group, and adult gooses (beyond one year) group.

### 2.3. RNA Extraction and Reverse Transcription PCR (RT-PCR)

Total RNA was extracted from fresh goose's small intestine and cecum tissue, using the RNA extract reagent TRIzol reagent (Invitrogen, Carlsbad, CA, USA), according to the manufacturer's instructions. The cDNA was synthesised using HiScript 1st Strand cDNA Synthesis Kit (Vazyme), according to the manufacturer's instructions. And cDNA templates of all different samples were stored at −70°C.

### 2.4. Cloning and Sequencing of goIFNLR1

The degenerate primers of goIFNLR1 were designed via multiple sequence alignments with the* Gallus gallus* IFNLR1 (chIFNLR1) (GenBank accession number FJ947118.1) and* Anas platyrhynchos* predicted IFNLR1 (duIFNLR1) (GenBank accession number XM_0050 28146.1); then the areas of high homology were selected to design primers and the partial sequence of goose IFNLR1 was amplified with the primers IFNLR1-1F and IFNLR1-1R. The procedure of PCR amplification is as follows: initial denaturation at 94°C for 5 min followed by 35 cycles at 94°C for 30 s, annealing at 54°C for 30 s and extension at 72°C for 20 s, and at the end of the thermal cycling, a further extension at 72°C for 10 min following the last cycle. The full-length cDNA sequences of goIFNLR1 were gained using 5′ and 3′ rapid amplification of cDNA ends (RACE) polymerase chain reactions (PCRs), and the gene-specific primers (GSPs) were designed according to the partial sequence of goIFNLR1 obtained (all the primers were listed in Table 1). The 5′-end and 3′-end of goIFNLR1 were obtained by using the Nested PCR technique to amplification. First of all, the full-length cDNAs should use specificity primers to reverse transcription; these primers were IFNLR1-5G0 and 3RACE-AP, respectively. Secondly, 5′-end of the synthesized first-strand cDNA should add a homopolymeric tail using TdT and dCTP (Beyotiome, China). However, for 3′-RACE, there was no need to add the homopolymeric. Thirdly, for 5′-RACE, the two rounds of Nested PCR using the primers of IFNLR1-5GSP1 with Abridged Anchor Primer (5RACE-AAP) and the primers IFNLR1-5GSP2 with Abridged Universal Amplification Primer (5RACE-AUAP), respectively. For 3′-RACE, the 3′-end of goIFNLR1 was amplified also by the method of Nested PCR using the primers of IFNLR1-3GSP1 with Abridged Universal Amplification Primer (3RACE-AUAP) and the primers IFNLR1-3GSP2 with Abridged Universal Amplification Primer (3RACE-AUAP). Then, the full-length cDNA sequence of goIFNLR1 was confirmed by using PrimeSTAR Max DNA Polymerase (TaKaRa Co., LTD, Japan) with the primers of IFNLR1-2F and IFNLR1-2R. The interest DNA product was purified by using the gel extraction kit (Tiangen, Beijing, China). Finally, PCR and RACE-PCR products were cloned into the pMD 19-T vector (TaKaRa Co., LTD, Japan), the selected clones were sequenced and analyzed by using the ABI 3730 XL sequencer (Applied Biosystems, Foster City, CA).

### 2.5. Quantitative Reverse Transcription PCR (qRT-PCR)

Bio-Rad CFX 96 Real-Time qPCR instrument was used for qRT-PCR, which was employed on total RNAs for detecting the tissue distribution of goIFNLR1 with primers of IFNLR1-3F and IFNLR1-3R (All the primers were listed in Table 1). 0.2 *μ*L cDNA from eleven different tissues was used as the template, 5 *μ*L EXPRESS SYBR Greener qPCR Super mix Universal (Invitrogen), 4.4 *μ*L ddH_2_O and 0.2 *μ*L intron-spanning primer which listed in Table 1. The cycling profile consisted of an initial denaturation at 95°C for 5 min, followed by 39 cycles of 95°C for 10 s, and 59°C for 30 s followed by melt curve analysis (from 65°C to 95°C with a heating rate at 0.5°C per second and a continuous fluorescence measurement). Expression level of the target genes was calculated using either standard curves method (goIFNLR1) or the comparative threshold cycle (Ct) method to derive fold change gene expression. Relative gene expression was calculated using the mean values obtained with the arithmetic formula ΔΔCt, as previously described. All data were normalized use the housekeeping gene goose beta-actin (GenBank accession number M26111.1) with the primers of *β*-actin-F and *β*-actin-R (Table 1) as an internal standard.

### 2.6. Bioinformation Analysis

The potential ORF of goIFNLR1 was analyzed by using open reading frames (ORF) finder program (http://www.ncbi.nlm.nih.gov/gorf/gorf.html), and the ORF region was translated into corresponding amino acids using DNAMAN software. The putative protein sequence of goIFNLR1 was compared with the other sequences by using the DNAMAN multiple sequence alignment. The signal peptides and transmembrane regions were predicted by SignalP 4.1 (http://www.cbs.dtu.dk/services/SignalP/) and TMHMM 2.0 servers (http://www.cbs.dtu.dk/services/TMHMM/), respectively. A phylogenetic tree of goIFNLR1 was constructed using the MEGA 6.0 neighbor-joining (NJ) method based on the other IFNLR1 amino acid sequences [19]; this software has 500 repetitions bootstrap analysis for the branches, which are represented by numbers at the branch nodes. The GenBank accession numbers of the sequences chosen for alignment were listed in Table 2. The percentage of similarity and divergence values between each sequence (goose and other thirteen bird species) were calculated through MegAlign program of the DNASTAR software package.

## 3. Results

### 3.1. Molecular Cloning and Characterization of goIFNLR1

**Figure 1:**
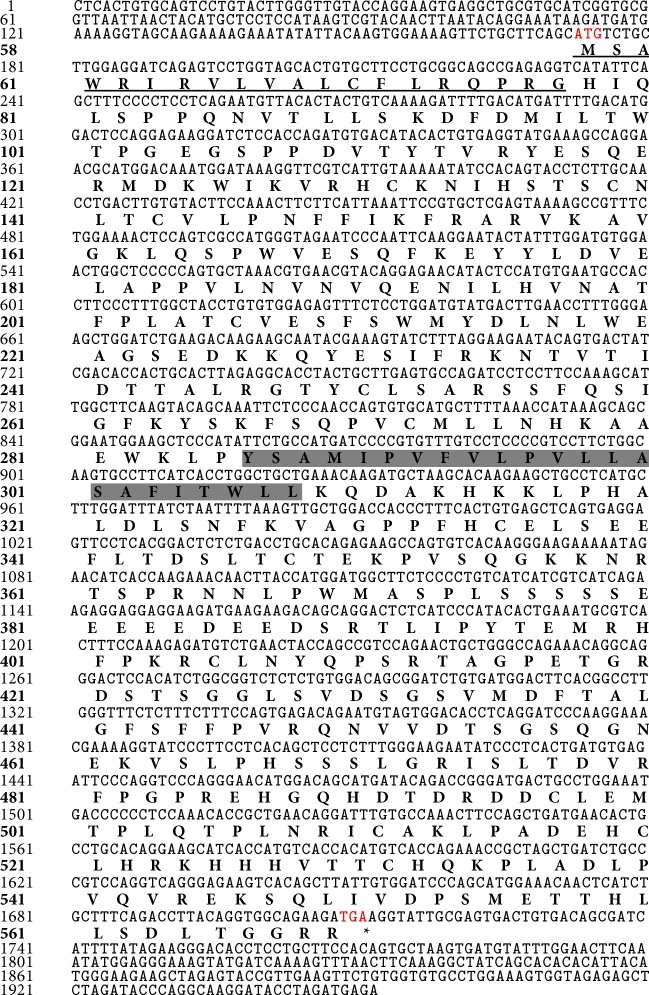
The nucleotides and deduced amino acid sequences of goIFNLR1 gene. The numbers of nucleotides were shown on the left and the numbers of amino acids were bold also on the left. The initiation codon and stop codon were displayed in red font, the predicted signal peptide of these genes was underlined, and the predicted transmembrane domain was shadowed.

**Figure 2:**
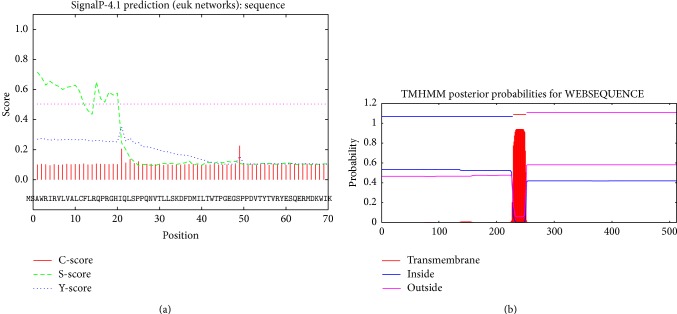
Predicted transmembrane region and signal peptide for goIFNLR1. (a) The signal peptide of goIFNLR1 was predicted by Signal 4.1 Server. C, S, and Y scores indicate cleavage sites, signal peptide, and combined cleavage site predictions, respectively. (b) The transmembrane region of goIFNLR1 was predicted by TMHMM 2.0 server, 23 aa were included in this region.

Geese are domesticated from* Anser* species, belong to the poultry, and have a high homology with other poultry, such as* Anas platyrhynchos, Gallus gallus,* and various kinds of birds. For the first time, here the identification of goIFNLR1 (GenBank accession number KP_232960) was reported. The full-length sequence of goIFNLR1 was 1952 bp and contains 172 bp 5′UTR and 241 bp 3′UTR (Figure 1). And the ORF of goIFNLR1 contained 1539 bp, encoding 512 amino acids (aa) with a 20 aa putative signal peptide (Figure 2(a)). Furthermore, there is a transmembrane region which includes 23 aa and transmembrane region separates the protein into a 228 aa intracellular domain and a 262 aa extracellular domain (Figure 2(b)).

The cDNA sequence of goIFNLR1 was highly conserved with that of other birds; the amino acid identity showed more than 90% with* Anas platyrhynchos* predicted IFNLR1 (duIFNLR1), whereas the amino acid identities decreased to less than 40% and 30% with that of mammalian and amphibian homologs, respectively (Table 2).

### 3.2. goIFNLR1 Gene Was Closely Related to Poultry IFNLR1 Genes

**Figure 3:**
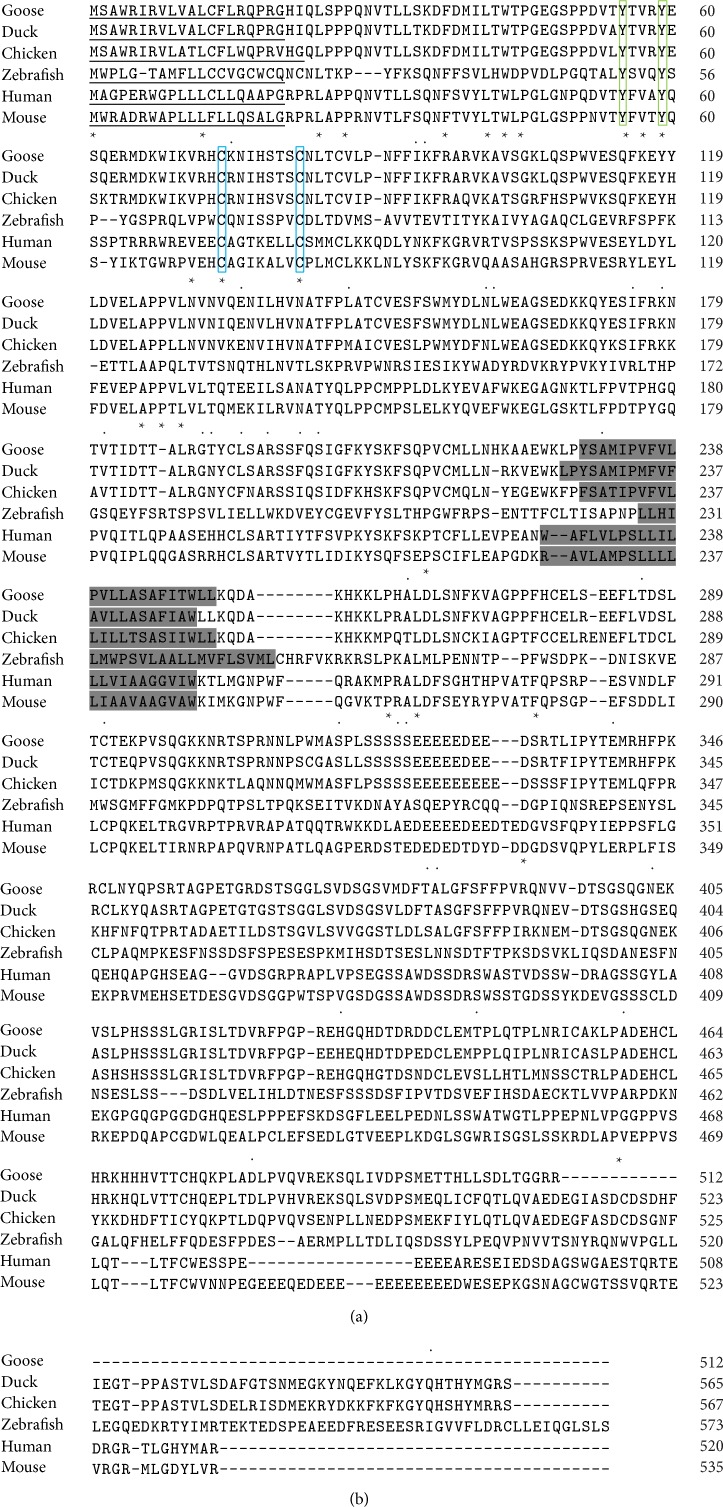
Multiple alignments of amino acid sequences of* Anser cygnoides, Anas platyrhynchos, Gallus gallus, Mus musculus, Homo sapien,* and* Danio rerio* IFNLR1. The sequences from other species were derived from GenBank with accession numbers XP_005028203.1 (*Anas platyrhynchos*), AHF20241.1 (*Gallus gallus*), NP_734464.1 (*Homo sapiens*), NP_777276.3 (*Mus musculus*), and NP_001184131.1 (*Danio rerio*). Alignment was generated with ClustalW 2. Amino acids conserved amongst all species were indicated as follows: (∗) identical and (.) weakly conserved. The predicted transmembrane sequences were indicated in gray shadow and the predicted signal peptide of these genes was underlined. Conserved cysteine residues and tyrosine were shown in green and blue box, respectively.

**Figure 4:**
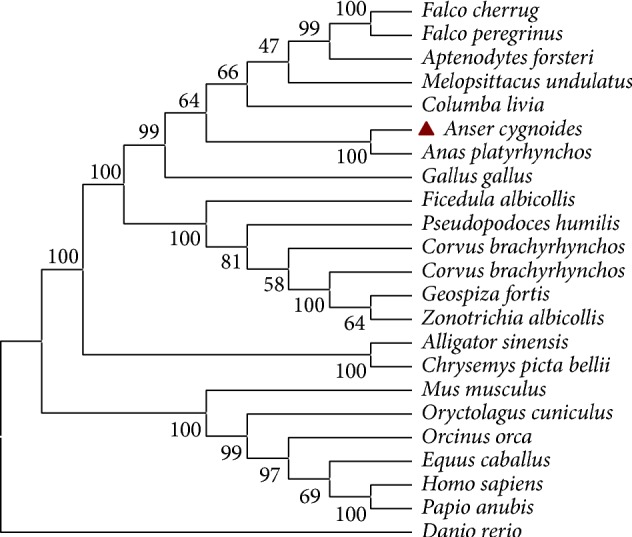
A phylogenetic tree of the amino acid sequences of IFNLR1 from different species. The putative gene-tree was built using the Neighbor-Joining method based on the alignment of IFNLR1 amino acid sequences. The percentage of replicate trees in which the associated taxa clustered together in the bootstrap test was shown next to the branches. Evolutionary analyses were conducted in MEGA 6.0. The sequences used were listed in Table 2.

The comparison of goIFNLR1 amino acid sequences with other reported IFNLR1 sequences (*Homo sapiens, Mus musculus, Gallus gallus, Anas platyrhynchos,* and* Danio rerio*) was illustrated in Figure 3. And the conserved intracellular region of goIFNLR1 protein presented two conserved cysteine residue (green box) and two conserved tyrosine in the intracellular region (blue box), respectively (Figure 3). The phylogenetic tree constructed using the amino acid sequences of the predicted goIFNLR1 proteins and other species was showed in Figure 4. Totally, thirteen putative amino acid sequences of birds IFNLR1 were aligned by MegAlign program of the DNASTAR software package. Among these species,* Anser cygnoides* IFNLR1 shows higher amino acid identity (>70%) values with the sequences of other five birds (poultry and falcon) and lower amino acid identity (<60%) values with finch IFNLR1 sequences (Table 3).

### 3.3. The Tissue Distribution of goIFNLR1

**Figure 5:**
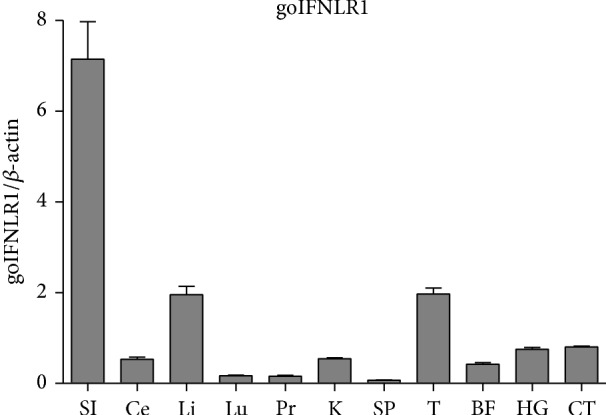
The qRT-PCR detection of IFNLR1 mRNA expression in different tissues from 3 healthy individuals. goIFNLR1 transcription was evaluated in small intestine (SI), cecum (Ce), liver (Li), lung (Lu), proventriculus (Pr), kidney (K), spleen (SP), thymus (T), bursa of fabricius (BF), harderian gland (HG), and cecal tonsil (CT). The error bars represented standard deviation. The goIFNLR1 mRNA was measured in qRT-PCR and normalized against the housekeeping gene (goose *β*-actin). Data were represented as the mean ± SEM (*n* = 4); the error bars indicate standard deviation.

To examine the tissue distribution of goose IFNLR1, Oligo(dT)_18_ was used as primer and the eleven tissues were used as templates to do reverse transcription PCR, the mRNA expression level of goIFNLR1 was determined by qRT-PCR. As shown in Figure 5, goIFNLR1 can be detected in all chosen tissues; the highest transcription was detected in the small intestine and the lowest transcription was detected in the spleen of 1W goose. Although a relatively high expression was also observed in the secondary immune organ (thymus, cecal tonsil, and harderian gland). Interestingly, there is a trend that goIFNLR1 has a high expression of level in epithelial cell (small intestine), which was consistent with what was previously reported.

### 3.4. The goIFNLR1 Expression Level in Epithelial Related Tissues

**Figure 6:**
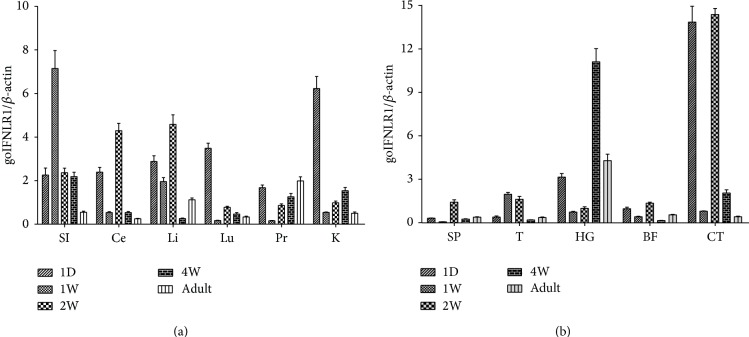
Expression of IFNLR1 in various goose tissues. (a) Expression of goIFNLR1 in the epithelial related tissues, including small intestine (SI), cecum (Ce), liver (Li), lung (Lu), proventriculus (Pr), and kidney (K) collected at the indicated time points, such as one-day-old gosling, one-week-old gosling, two-week-old gosling, four-week-old gooses, and adult gooses (beyond one year). (b) Expression of goIFNLR1 in the immune tissues, including spleen (SP), thymus (T), harderian gland (HG), bursa of fabricius (BF), and cecal tonsil (CT); goIFNLR1 mRNA was measured in qRT-PCR and normalized against the housekeeping gene (goose *β*-actin). Data were represented as the mean ± SEM (*n* = 4); the error bars indicate standard deviation.

Considering the ubiquitous distribution of goIFNLR1 was observed, we are wondering if the expression of goIFNLR1 was changed in an age-dependent way in the epithelial related tissues. Goose primary epithelial related tissues derived from small intestine, kidney, liver, lung, proventriculus, and cecum were collected for the detection of goIFNLR1 expression by qRT-PCR. As shown in Figure 6(a), small intestine has a high and a relatively stable expression in all age groups, specially, in goslings (one-week-old). Moreover, a relative high expression of goIFNLR1 was detected in the epithelial related tissues of one-day-old goslings, specially, in kidney. The expression level of goIFNLR1 in the lung and kidney decreased in an age-dependent way, and the lowest expression level was shown in the lung, kidney, and proventriculus of one-week-old goslings. However, surprisingly a relative low expression of goIFNLR1 was observed in the epithelial related tissues of geese (four-week-old geese and adult geese).

### 3.5. The goIFNLR1 Expression in Immune Defense Related Tissues

Five immune tissues, including spleen, Cecal tonsil, harderian gland, thymus, and bursa of fabricius, were collected for the detection of goIFNLR1 expression by qRT-PCR. As shown in Figure 6(b) the highest expression level of goIFNLR1 was detected in the cecal tonsil of one-day-old and two-week-old geese and in the harderian gland of four-week-old geese. And the tissues spleen and thymus always show the low goIFNLR1 expression level in all age groups. Specially, the lowest expression level of goIFNLR1 was detected in the spleen of one-week-old birds, as well as in the thymus and bursa of fabricius of four-week-old geese.

## 4. Discussion

China has the largest breeding population of waterfowl in the world, and aquatic birds are the natural reservoirs for many viruses, including many that cause significant morbidity and mortality, such as avian influenza virus (AIV), and are in charge of the transmission and dissemination of pathogens [20]. Goose, as avian influenza virus carrier, shows no clinical signs [21] that might due to some different immune characters exist. IFNs play an important role in innate antiviral immune response, and type III IFNs were newly discovered in 2003 [3]. The antiviral activity of IFN-*λ* is similar to type I IFNs [6]. In the case of rotavirus infection, IFN-*λ* plays a much stronger defense effect than type I interferon [22, 23]. Amusingly, in human and mice the type III interferon receptor 1 has a restricted tissue distribution and predominantly in epithelial cell [17, 24]. Possibly this may be the reason why IFN-*λ* has a primarily protection of mucosal entities. And IFN-*λ* was recognized by its receptors to active the subsequently reaction. Moreover, the recognition between IFN-*λ* and its receptor is the first and most important step to activate the host's antiviral immune response.

Towards a better understanding of goose type III interferon system, we described goIFNLR1 cDNA from the Sichuan White Goose for the first time in this study. The protein sequence of goIFNLR1 was highly conserved with the sequence from other avian especially with duck (90%) (Table 2 and Figures 3 and 4). Furthermore, a phylogenetic tree was performed, which indicated goIFNLR1 has a high homology with duck and other birds (Figure 4). Additionally, the predicted signal peptide region was conserved with duck, chicken, mouse, and human (Figure 3). And a transmembrane domain of goIFNLR1 was predicted, which can demonstrate that goIFNLR1 was a single membrane protein (Figure 3).

There are considerable differences in the tissue distribution characters between IFNAR and IFNLR1. It is reported IFNAR shows a ubiquitously expression. However, in mammals and amphibians such as human, bat, and* Xenopus*, IFNLR1 has a restricted expression with a high level in the lung, heart, stomach, and spleen and a low level in the brain [5, 15, 24]. In mice, IFNLR1 was mainly expressed in the small intestine, stomach, and skin and has shown the lowest expression level in immune organ (spleen) [17, 25, 26]. In chicken, chIFNLR1 transcription level has shown a low expression in brain and a high expression in lung, intestine, and liver [10]. Our results were consistent with some of these conclusions; the expression of goIFNLR1 is ubiquitous in all tested tissues of Chinese goose. Moreover, similar to chicken, a relatively high expression of goIFNLR1 was observed in small intestine and liver (epithelial related tissues), as well as in thymus (the immune organs). While similar to mice, the lowest expression level of it was detected in the spleen. As the report, liver is a virus targeted tissue with a large number of hepatocytes, and human hepatocyte cell lines indeed express the IFNLR1 and readily respond to IFN-*λ* [27–29]. Accordingly, IFN-*λ* was shown to restrict HCV replication in hepatoma cell lines [27]. Although spleen is an important immune tissue, our result suggested that a low expression level of IFNLR1 gene was detected in spleen, which is maybe because the spleen was composed of lots of immunocytes not epithelia.

For a better understanding of the developmental expression of goIFNLR1 in vivo, the qRT-PCR detection of goIFNLR1 gene in epithelial related tissues and immune defense related tissues of five age brackets (one-day, one-week, two-week, four-week, and adult) were accessed here. In various groups, the epithelial related tissues have a relatively high expression of goIFNLR1 when compared to the immune tissues. Among all epithelial related tissues, small intestine shows a higher expression level of goIFNLR1 during the whole developmental stage, and expression level peaked at the one-week time point. One of the reasons is maybe related to the maternally transmitted resistance. Many investigators reported that the immune system of young birds was inherited by their master tape in embryonic period [30, 31]. This phenomenon was also reflected in other tissues cecum, liver, lung, and kidney. Proventriculus also belongs to the epithelial related tissue; the expression level of IFNLR1 in proventriculus dropped to the lowest level in one week then gradually increased. Perhaps because IFN-*λ* was secreted by epithelial cells that mainly are located in respiratory and gastrointestinal tracts [23, 32], the high expression of goIFNLR1 was observed in the epithelial related tissues, especially in young birds.

Further investigation of goIFNLR1 expression level in the immune tissues found that goIFNLR1 has a relatively high expression level in harderian gland and cecal tonsil compared to other immune tissues. The possible explanation was both cecal tonsil and harderian gland are open immune tissues, the group as mucosa-associated lymphoid tissue that play a critical role against the virus invasion. Moreover, a lower expression level was shown in the spleen, thymus, and bursa of fabricius; one reason may be the immune tissues (thymus and bursa of fabricius) have a degradation with the growth of age in birds.

To our knowledge, this is the first report of the molecular cloning and the feature description of IFNLR1 in the goose, demonstrating that goIFNLR1 has a restricted tissue distribution mainly in epithelial related tissues during the developmental period. This result conform to the tissues distribution character of IFNLR1 gene of other species, and it may shed a light on the type III interferon system mediated antiviral immune responses in aquatic birds. Indeed, further more experiments are needed to better understand the interaction between IFN-*λ* and its receptors in goose.

## Figures and Tables

**Table 1:** The list of primers.

Primer name	Sequence 5′-3′	Application
Oligo(dT)_18_	TTTTTTTTTTTTTTTTTT	Reverse transcription
IFNLR1-1F	ACATGG(A/G)C(T/G)CC(A/G)GGAGAAGG	Degenerate PCR
IFNLR1-1R	TCTTGTCTTCAGA(T/C)CC(G/A/T/C)GC	Degenerate PCR
IFNLR1-5G0	AGGCGGCATTCAC	5RACE PCR
IFNLR1-5GSP1	AGCCAGTTCCACATCCAAAT	5RACE PCR
IFNLR1-5GSP2	TCCTGGCTTTCATACCTCAC	5RACE PCR
5RACE-AAP	GGCCACGCGTCGACTACGGGIIGGGIIGG GIIGGGIIG	5RACE PCR
5RACE-AUAP	GGCCACGCGTCGACTAGTAC	5RACE PCR
IFNLR1-3GSP1	CTATTTGGATGTGGAACTGGCTC	3RACE PCR
IFNLR1-3GSP2	CCTGGATGTATGACTTGAACC	3RACE PCR
3RACE-AP	GGCCACGCGTCGACTAGTACT(17)	3RACE PCR
3RACE-AUAP	GGCCACGCGTCGACTAGTAC	3RACE PCR
IFNLR1-2F	ATGCTCCTCCATAAGTCG	Full-length amplification
IFNLR1-2R	GCTGTGACTTCTCCCTGA	Full-length amplification
IFNLR1-3F	TCTGAAGACAAGAAGCAAT	qRT-PCR
IFNLR1-3R	GCACACTGGTTGGGAGAAT	qRT-PCR
*β*-actin-F	CCGTGACATCAAGGAGAA	qRT-PCR
*β*-actin-R	GAAGGATGGCTGGAAGAG	qRT-PCR

**Table 2:** Taxonomy of species, accession numbers of IFNLR1 sequences, and the amino acid similarity and identity with goIFNLR1 proteins were analyzed using BLASTP with a BLOSUM62 scoring matrix. Identity means complete identical amino acid pairs.

Taxonomy	Species	*Anser cygnoides *	Accession number
		Identity (%)	
Birds	*Anas platyrhynchos *	90	XP_005028203.1
	*Gallus gallus *	73	AHF20241.1
	*Ficedula albicollis *	63	XP_005058494.1
	*Pseudopodoces humilis *	58	XP_005529866.1
	*Falco cherrug *	75	XP_005433108.1
	*Falco peregrinus *	74	XP_005234192.1
	*Melopsittacus undulatus *	70	XP_005142513.1
	*Columba livia *	68	XP_005513512.1
	*Corvus brachyrhynchos *	61	XP_008633390.1
	*Serinus canaria *	62	XP_009096915.1
	*Geospiza fortis *	58	XP_005427215.1
	*Zonotrichia albicollis *	57	XP_005494786.1
	*Aptenodytes forsteri *	80	XP_009274512.1

Reptiles	*Alligator sinensis *	47	XP_006019447.1
	*Chrysemys picta bellii *	48	XP_005302269.1

Mammalia	*Mus musculus *	36	NP_777276.3
	*Oryctolagus cuniculus *	32	XP_002716066.1
	*Orcinus orca *	35	XP_004266740.1
	*Equus caballus *	37	XP_005607410.1
	*Homo sapiens *	34	NP_734464.1
	*Papio Anubis *	35	XP_003891375.2

Fish	*Danio rerio *	26	NP_001184131.1

**Table 3:** Similarity of IFNLR1 sequences between goose and other birds analyzed by MegAlign. The percentage of similarity was showed in upper triangle and the percentage of divergence was showed in lower triangle.

Species	1	2	3	4	5	6	7	8	9	10	11	12	13
(1) *Anas platyrhynchos *	∗∗∗	89.5	67.7	60.1	73.8	73.6	60.3	71.6	58.1	67.3	56.7	58.6	57.3
(2) *Anser cygnoides *	11.4	∗∗∗	68.5	61.8	77.1	76.9	62.2	73.0	59.8	70.1	58.9	58.7	58.9
(3) *Columba livia *	42.1	40.9	∗∗∗	63.1	77.4	77.2	60.8	63.6	61.0	70.5	60.6	61.6	60.2
(4) *Corvus brachyrhynchos *	56.3	52.9	50.4	∗∗∗	67.9	67.9	81.8	59.0	85.0	65.0	83.4	83.0	81.9
(5) *Falco cherrug *	32.3	27.4	26.9	41.7	∗∗∗	99.8	66.0	72.1	65.0	76.1	64.5	64.4	63.4
(6) *Falco peregrinus *	32.6	27.7	27.2	41.7	0.2	∗∗∗	66.0	71.9	65.0	75.9	64.5	64.4	63.4
(7) *Ficedula albicollis *	56.0	52.3	54.9	20.9	45.1	45.1	∗∗∗	57.1	84.6	63.4	81.4	81.3	82.6
(8) *Gallus gallus *	35.8	33.4	49.6	58.6	34.9	35.2	62.6	∗∗∗	57.3	65.5	55.3	56.1	55.9
(9) *Geospiza fortis *	60.5	56.9	54.6	16.7	46.9	46.9	17.3	62.6	∗∗∗	62.4	83.1	90.3	92.1
(10) *Melopsittacus undulatus *	2.8	38.0	37.5	46.8	28.9	29.1	49.9	46.0	51.8	∗∗∗	61.7	61.9	60.7
(11) *Pseudopodoces humilis *	63.5	58.9	55.3	18.8	47.8	47.8	21.5	66.8	19.1	53.2	∗∗∗	80.9	80.9
(12) *Serinus canaria *	59.5	59.2	53.4	19.4	48.0	48.0	21.6	65.0	10.4	52.7	22.1	∗∗∗	89.0
(13) *Zonotrichia albicollis *	62.3	58.8	56.3	20.8	49.8	49.8	19.9	65.3	8.4	55.2	22.1	11.9	∗∗∗
